# Comparison of time to failure of pembrolizumab plus chemotherapy versus pembrolizumab monotherapy: a consecutive analysis of patients having NSCLC with high PD-L1 expression

**DOI:** 10.1007/s00262-021-03029-9

**Published:** 2021-08-14

**Authors:** Hiroshi Takumida, Hidehito Horinouchi, Ken Masuda, Yuki Shinno, Yusuke Okuma, Tatsuya Yoshida, Yasushi Goto, Noboru Yamamoto, Yuichiro Ohe

**Affiliations:** Department of Thoracic Oncology, National Cancer Center Hospital, 5-1-1 Tsukiji, Chuo-ku, Tokyo, 104-0045 Japan

**Keywords:** Non-small cell lung cancer, Immune checkpoint inhibitors, Drug therapy, Pembrolizumab, PD-L1, Time to failure of strategy

## Abstract

**Introduction:**

There are two treatment strategies for non-small cell lung cancer (NSCLC) exhibiting a high expression level of programmed death-ligand 1 (tumor proportion score ≥ 50%): pembrolizumab plus chemotherapy and monotherapy. We retrospectively compared their efficacy and safety.

**Materials and methods:**

We reviewed the efficacy and safety of first-line pembrolizumab-containing regimens administered between 2017 and 2020 to consecutive patients. The patients were divided into a pembrolizumab plus chemotherapy group (Combo group) or monotherapy group (Mono group). To compare the efficacy, we monitored the time to failure of strategy (TFS) defined as the time from the start of treatment to the occurrence of one of the following events: the addition of any drug not included in the primary strategy, progression of cancer after complete therapy, progression and no subsequent therapy, or death, whichever occurred first. We used the propensity score matching (PSM) to reduce the bias.

**Results:**

A total of 126 patients were identified (89 in the Mono group and 37 in the Combo group). PSM matched 36 individuals from each of the two groups. The overall response rate and median progression-free survival of the Combo group were better than those of the Mono group. However, the median TFS was almost the same (11.3 months vs. 14.9 months; hazard ratio 1.40 [95% confidence interval 0.62–3.15]). The frequency of all serious adverse effects was higher in the Combo group than in the Mono group.

**Discussion:**

Due to similar efficacy in TFS, both pembrolizumab plus chemotherapy and monotherapy are valid options for NSCLC.

**Supplementary Information:**

The online version contains supplementary material available at 10.1007/s00262-021-03029-9.

## Introduction

Several trials have demonstrated the efficacy of pembrolizumab, an immune checkpoint inhibitor, in patients with advanced non-small cell lung cancer (NSCLC) exhibiting a high expression level of programmed death-ligand 1 (PD-L1) (tumor proportion score [TPS] ≥ 50%) [1–4]. For example, the KEYNOTE-024 trial showed that pembrolizumab monotherapy was effective for patients with TPS > 50%, regardless of the histology [1]. The subset analysis of the KEYNOTE-042 trial, in which pembrolizumab was administered to patients with TPS > 1%, also showed consistent results [2]. In other trials that evaluated the efficacy of the addition of pembrolizumab to platinum doublet chemotherapy, the combination therapy was superior to chemotherapy alone in a subset analysis of the KEYNOTE-189 trial and the KEYNOTE-407 trial, respectively. The KEYNOTE-189 trial showed the efficacy of cisplatin or carboplatin + pemetrexed (PEM) + pembrolizumab in non-squamous cell carcinoma [3]. The KEYNOTE-407 trial showed the efficacy of platinum + (nanoparticle albumin-bound [nab-]) paclitaxel (PTX) + pembrolizumab in patients with squamous cell carcinoma [4].

However, there is no clear consensus on whether pembrolizumab monotherapy followed by chemotherapy or combined pembrolizumab plus chemotherapy is superior for the treatment of advanced NSCLC with PD-L1 TPS ≥ 50%. Moreover, there has been only an indirect comparison between the two strategies. For example, in the 4-year follow-up data of the KEYNOTE-189 trial, the 3-year survival rate of the TPS high population was 43.7% [5], which is comparable with the 3-year survival rate (43.7%) in the analysis of the 5-year follow-up data of the KEYNOTE-024 trial [6]. There are other two reports used real-world data; however, the trend was similar [7, 8]. The significance of combining chemotherapy is not clear when comparing these results.

To address these questions, we retrospectively compared the efficacy and safety of pembrolizumab monotherapy and pembrolizumab plus platinum doublet therapy.

## Materials and methods

### Study design and patients

We consecutively reviewed patients with NSCLC (aged ≥ 18 years) with a PD-L1 TPS ≥ 50% who received first-line treatment with pembrolizumab between 2017 and 2020 at the National Cancer Center Hospital, Japan. The patients were divided into two groups: a pembrolizumab monotherapy group (Mono group) and a combined pembrolizumab plus chemotherapy group (Combo group). We then evaluated the TFS, which was defined as the time from the start of treatment to the occurrence of one of the following events: the addition of any drug not in the primary strategy, progression of cancer after complete therapy, progression and no subsequent therapy, or death, whichever occurred first. Moreover, we evaluated the objective response rate (ORR), progression-free survival (PFS), overall survival (OS), and safety.

To reduce the bias in the choice of regimen, we excluded patients who had been previously treated with chemotherapy, such as perioperative chemotherapy, chemoradiotherapy, or those who received pembrolizumab in the clinical trial. We also excluded patients with confirmed positive driver oncogenes. Furthermore, we used the propensity score matching (PSM) method to reduce the selection bias due to the patient background.

### Data collection

The value of PD-L1 TPS was defined as described in a previous clinical trial [1]. All values were determined using the PD-L1 IHC 22C3 pharmDx assay (Agilent Dako). Efficacy was determined according to the New Response Evaluation Criteria in Solid Tumors: Revised RECIST guideline (version 1.1) [9]. Adverse events (AEs), including abnormalities in the results of laboratory investigations, were graded according to the National Cancer Institute Common Terminology Criteria for Adverse Events, version 5.0 [10] until the beginning of the second line of treatment. Serious adverse events (SAEs) were defined as AEs resulting in death or risk of death, hospitalization for treatment, or prolonged hospitalization. The cut-off date was April 30, 2021.

### Statistical analysis

PSM was applied at a ratio of 1:1 with a caliper width equal to 0.2 of the standard deviation of the logit of the propensity score to compare the pembrolizumab monotherapy group and the pembrolizumab plus chemotherapy group. Factors, such as age, sex, smoking history, PD-L1 TPS, history of lung disease, performance status, histology, and staging, were matched.

The follow-up period, TFS, PFS, and OS were estimated using the Kaplan–Meier method. Data of the patients who were alive or lost to follow-up were censored for OS when they were last known to be alive. Data of patients who were alive and did not have disease progression or those who were lost to follow-up were censored for the analysis of PFS or TFS at the time of the last imaging assessment.

The hazard ratios (HRs) and 95% confidence intervals (CIs) were calculated using the Cox proportional hazards model. HR and 95% CI after PSM were calculated using the stratified Cox proportional hazards model. All statistical analyses were performed using the EZR (Easy R) statistical software version 1.54 [11]. Statistical significance was set at *p* < 0.05.

## Results

### Patient selection

**Fig. 1:**
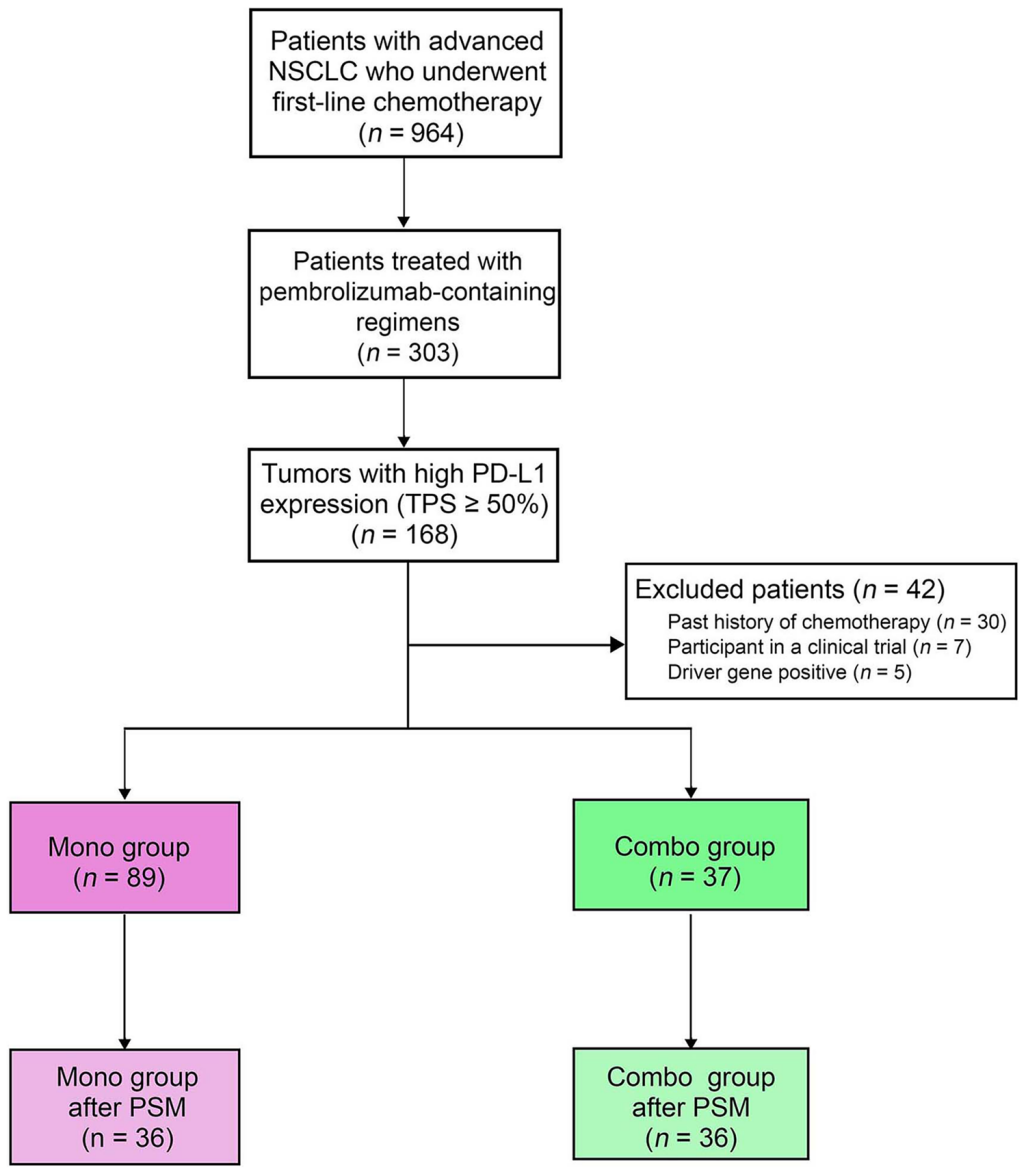
Patient selection. Analysis of the data of patients with advanced-stage NSCLC from 2017 to 2020 was performed. Eighty-nine patients were assigned to the Combo group, and 37 patients were assigned to the Mono group. PSM matched 36 people from each of the two groups. NSCLC, non-small cell lung cancer; PD-L1, programmed death-ligand 1; TPS, tumor proportion score; PSM, propensity score matching

Of the 964 patients with advanced NSCLC who underwent first-line treatment, 303 patients received a pembrolizumab-containing regimen. The number of patients with a PD-L1 high TPS was 168. Forty-two patients were excluded from the study. Therefore, 126 patients were included in the analysis. The pembrolizumab monotherapy group comprised 89 patients, and the combined pembrolizumab plus chemotherapy group comprised 37. The PSM matching identified 36 individuals from each of the two groups (Fig. 1). The median follow-up period of the pembrolizumab monotherapy group was 30.3 months (range 0.1–40.8 months), and that of the combined pembrolizumab plus chemotherapy group was 19.2 months (range 2.3–27.9 months).

### Patients’ characteristics (Table1)

**Table 1:** Patients’ characteristics

Characteristics	All groups (*n* = 126)	Mono group (*n* = 89)	Mono group after PSM (*n* = 36)	Combo group (*n* = 37)	Combo group after PSM (*n* = 36)
Regimen, n (%)					
Pembrolizumab	89(70.6)	89(100.0)	36(100.0)	0(0.0)	0(0.0)
Platinum + PEM + Pembro	30(23.8)	0(0.0)	0(0.0)	30(81.1)	30(83.3)
Platinum + (nab-) PTX + Pembro	7(5.6)	0(0.0)	0(0.0)	7(18.9)	6(17.7)
Age in years, median (range)	68.5(38–94)	69(38–94)	66(45–80)	64(46–78)	64(46–78)
Smoking status, n (%)					
Current or former	107(84.9)	76(85.3)	32(88.9)	31(83.8)	30(83.3)
Never	19(15.1)	13(14.6)	4(11.1)	6(16.2)	6(17.7)
Male sex, n (%)	92(73.0)	65(73.0)	27(75.0)	27(73.0)	27(75.0)
Performance status, *n* (%)					
0 or 1	97(77.0)	65(73.0)	33(91.7)	32(86.5)	31(86.1)
≥ 2	29(23.0)	24(27.0)	3(8.3)	5(13.5)	5(13.9)
History of lung disease, *n* (%)	57(45.2)	46(51.7)	15(41.6)	11(29.7)	11(30.6)
Stage, *n* (%)					
IV	89(70.6)	55(61.8)	33(91.7)	34(91.9)	33(91.7)
Recurrence Other	31(24.6) 6(4.8)	28(31.5) 6(6.7)	3(8.3) 0(6.7)	3(8.1) 0(0.0)	3(8.3) 0(0.0)
Histology, *n* (%)					
Ad	77(61.1)	51(57.3)	23(63.9)	26(70.3)	26(72.2)
Sq	25(19.8)	19(21.3)	3 (8.3)	6(16.2)	5(13.9)
Other	24(19.0)	19(21.3)	10(27.8)	5(13.5)	5(13.9)
Tumor PD-L1 status, *n* (%)					
TPS ≥ 75%	86(68.3)	60(67.4)	29(80.1)	26(63.4)	26(72.2)
75% > TPS ≥ 50%	40(31.8)	29(32.6)	7(19.4)	11(26.8)	10(27.8)

*Ad*, adenocarcinoma; *PD-L1*, programmed cell death-ligand 1; *PEM*, pemetrexed; Pembro, pembrolizumab, *PSM*, propensity score matching; *PTX*, paclitaxel; *nab-PTX*, nanoparticle albumin-bound paclitaxel; *Sq*, squamous cell carcinoma; *TPS*, tumor proportion score

The regimen of platinum + PEM + pembrolizumab was administered to 30 patients (81.1%) in the combined pembrolizumab plus chemotherapy group, and the regimen of platinum + (nab-) PTX + pembrolizumab was administered to seven patients (18.9%). Patients in the pembrolizumab monotherapy group were older and had worse performance status at baseline (PS), and a higher proportion of patients had a history of lung disease. The percentage of patients with relapse of the disease was higher in the pembrolizumab monotherapy group than in the pembrolizumab plus chemotherapy group. We used PSM to adjust for factors that differed between groups or were clinically important; PSM allowed the pembrolizumab monotherapy group and the pembrolizumab plus chemotherapy group to be almost identical in age, PS, and staging. The number of patients with history of lung diseases decreased in the pembrolizumab monotherapy group after PSM.

### First-line treatment

**Fig. 2:**
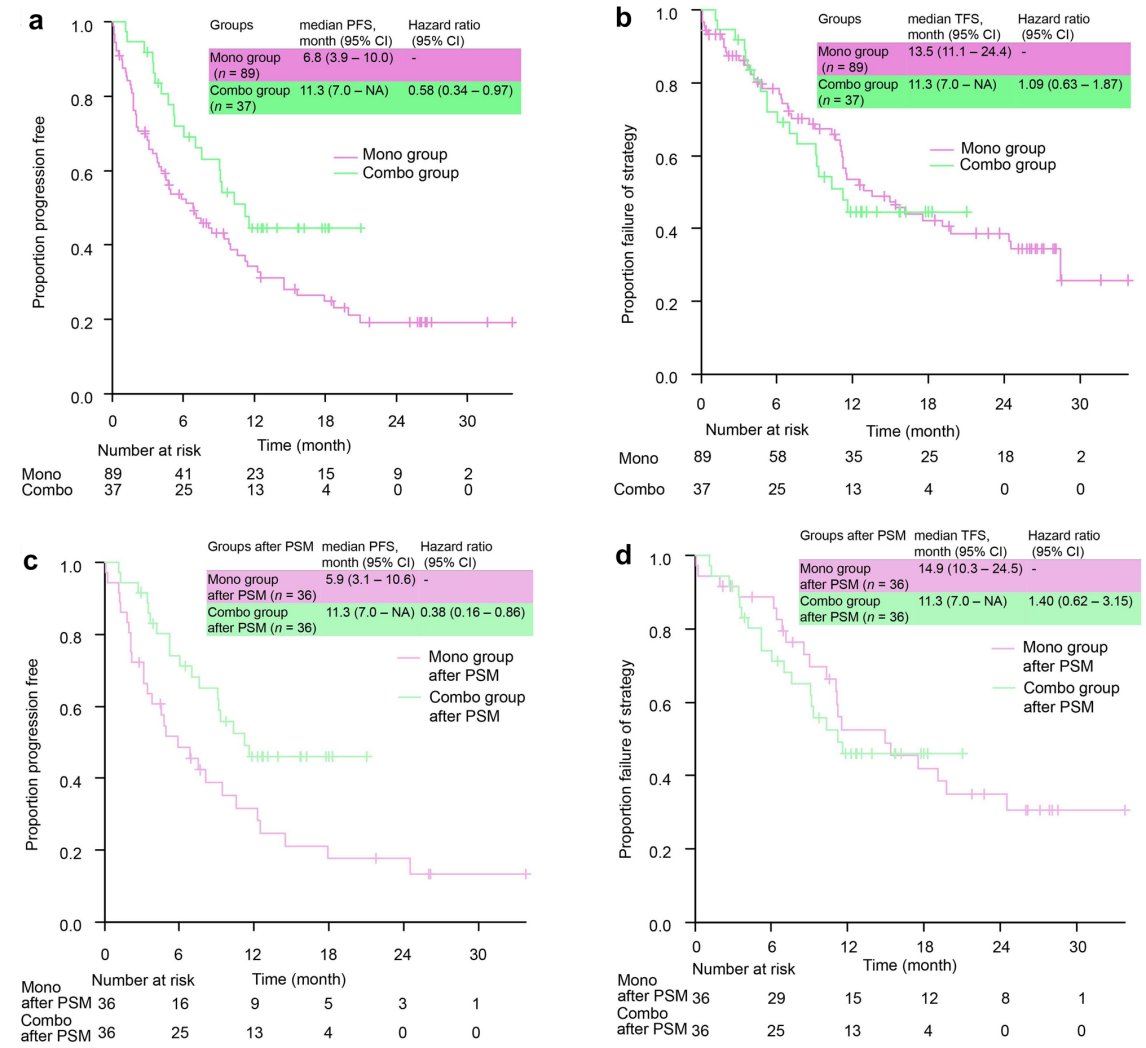
Survival curve **a** Progression-free survival, **b** Time to failure of strategy, **c** Progression-free survival after PSM, **d** Time to failure of strategy after PSM. PFS and TFS were estimated using the Kaplan–Maier method. PFS was significantly longer in the pembrolizumab plus chemotherapy group than in the pembrolizumab monotherapy group, and this trend did not change after PSM (HR 0.38 [95%CI 0.16–0.86], stratified Cox proportional hazards model). TFS was almost the same and this trend did not change after PSM, too (HR 1.40 [95% CI 0.62–3.15], stratified Cox proportional hazards model). PSM, propensity score matching; PFS, progression-free survival; TFS, time to failure of strategy; CI, confidence interval; NA, not applicable

The ORR was 43.8% in the pembrolizumab monotherapy group and 67.6% in the pembrolizumab plus chemotherapy group. The disease control rate was 64.0% in the pembrolizumab monotherapy group and 94.6% in the pembrolizumab plus chemotherapy group. These results did not change after PSM (supplementary Table 1). The median PFS (mPFS) was also significantly longer in the pembrolizumab plus chemotherapy group than in the pembrolizumab monotherapy group (11.3 months vs. 6.8 months; HR 0.58 [95% CI 0.34–0.97]) (Fig. 2a), and these results did not change after PSM (Fig. 2b).

### Time to failure of strategy (TFS)

To compare the efficacy of the two treatments, we used TFS, which was defined as the time from the start of treatment to the occurrence of one of the following events: the addition of any drug not in the primary strategy, progression of cancer after complete therapy, progression and no subsequent therapy, or death, whichever occurred first.

In contrast to the PFS, the median TFS (mTFS) was not significantly different between the two groups (11.3 months vs. 13.5 months; HR 1.09 [95% CI 0.63–1.87]) (Fig. 2c), and these trends did not change after PSM (11.3 months vs. 14.9 months; HR 1.40 [95% CI 0.62–3.15]) (Fig. 2d).

### Second-line treatment in the pembrolizumab monotherapy group

In the pembrolizumab monotherapy group, the proportion of patients who received second-line treatment after disease progression was 49.2% (31 out of 63 patients). A platinum-containing regimen was administered to 74% of patients who received second-line treatment (23 patients). In regimens, including PEM, 72% of patients transitioned to maintenance therapy. The ORR of second-line treatment was 41.9% (13 patients of 31). After PSM, the proportion of patients who received second-line treatment after disease progression was 67.9% (19 patients of 28), and a platinum-containing regimen was used in 63.1% of patients who received second-line treatment (12 patients). The ORR of the second regimen was 42.1% (8 patients of 19). After PSM, there was an increase in the proportion of patients who received second-line treatment after pembrolizumab treatment. However, the efficacy of the treatment did not change.

### Overall survival

**Fig. 3:**
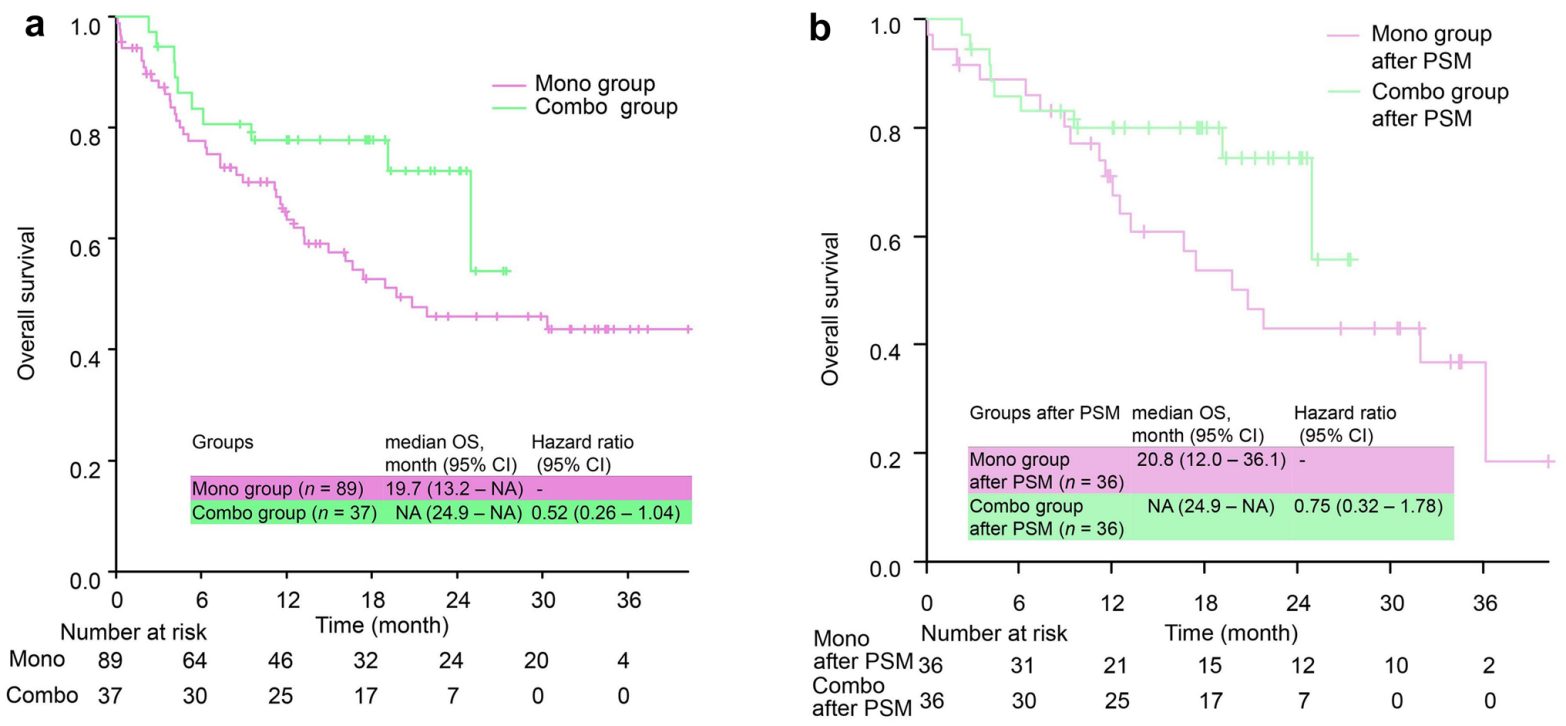
Overall survival, **a** Overall survival of the two groups. **b** Overall survival after PSM. OS was estimated using the Kaplan–Maier method. However, the data are immature because of the large number of censored patients. The median OS in the Pembro + Chemo group tended to be slightly better, but the difference narrowed after PSM (HR 0.75 [95% CI 0.32 – 1.78], stratified Cox proportional hazards model). PSM, propensity score matching; OS, overall survival; CI, confidence interval; NA, not applicable

The median OS was not different between the groups (Fig. 3a), and it was consistent after PSM (Fig. 3b). However, OS was immature because only 27.0% (10 patients) in the pembrolizumab plus chemotherapy group died in the observation period. In contrast, 47.1% (42 patients) in the pembrolizumab monotherapy group died. For the efficacy, i.e., ORR, PFS, TFS, and OS, subgroup analysis was performed by histological type, whether it was squamous cell carcinoma, but the trend did not change significantly (supplementary Table 2 and supplementary Fig. 1). However, subset analysis in squamous cell carcinoma should be evaluated cautiously due to substantially small number of patients with squamous histology.

### Adverse events (Table 2)

**Table 2:** Adverse events

Event	Mono group (*n* = 89)		Mono group after PSM (*n* = 36)		Combo group (*n* = 37)		Combo group after PSM (*n* = 36)	
	Any grade	Grade 3 or 4	Any grade	Grade 3 or 4	Any grade	Grade 3 or 4	Any grade	Grade 3 or 4
Number of patients (%)								
Any event (not irAE)	88(98.9)	48(53.9)	36(100.0)	22(61.1)	37(100.0)	27(73.0)	36(100.0)	26(72.2)
WBC count decreased	0(0.0)	0(0.0)	0(0.0)	0(0.0)	26(70.3)	9(24.3)	25(69.4)	9(25.0)
Neutrophil count decreased	0(0.0)	0(0.0)	0(0.0)	0(0.0)	24(64.9)	14(37.8)	24(66.7)	14(38.9)
Lymphocyte count decreased	35(39.3)	10(11.2)	13(36.1)	4(11.1)	22(59.5)	11(29.7)	21(58.3)	11(30.6)
Febrile neutropenia	0(0.0)	0(0.0)	0(0.0)	0(0.0)	5(13.5)	5(13.5)	5(13.9)	5(13.9)
Anemia	32(36.0)	8(9.0)	13(36.1)	2(5.6)	32(86.5)	11(29.7)	31(86.1)	11(30.6)
Platelet count decreased	12(13.5)	2(2.2)	4(11.1)	0(0.0)	26(70.3)	6(16.2)	26(72.2)	6(16.7)
Constipation	18(20.2)	0(0.0)	7(19.4)	0(0.0)	31(83.8)	0(0.0)	30(83.3)	0(0.0)
Anorexia	24(27.0)	3(3.4)	10(27.8)	0(0.0	28(75.7)	2(5.4)	28(77.8)	2(5.6)
Nausea	22(24.7)	1(1.1)	12(33.3)	0(0.0)	23(62.1)	1(2.7)	23(63.9)	1(2.8)
Malaise	36(40.4)	4(4.5)	15(41.7)	2(5.6)	21(56.8)	1(2.7)	21(58.3)	1(2.8)
AST increased	42(47.2)	4(4.5)	19(52.8)	3(8.3)	28(75.7)	2(5.4)	28(77.8)	2(5.6)
ALT increased	41(46.0)	4(4.5)	16(44.4)	3(8.3)	23(62.1)	1(2.7)	22(61.1)	1 (2.8)
Hypoalbuminemia	44(49.4)	10(11.2)	18(50.0)	2(5.6)	22(59.5)	2(5.4)	21(58.3)	2(5.6)
Creatinine increased	23(25.8)	0(0.0)	9(25.0)	0(0.0)	15(40.5)	0(0.0)	14(38.9)	0(0.0)
Any irAE	64(71.9)	13(14.6)	28(77.8)	8(22.2)	26(70.3)	4(10.8)	25(69.4)	4(11.1)
Eczema	42(47.2)	2(2.2)	20(55.6)	1(2.8)	18(48.6)	0(0.0)	17(47.2)	0(0.0)
Pneumonitis	22(24.7)	5(5.6)	9(25.0)	2(5.6)	4(10.8)	1(2.7)	4(11.1)	1(2.8)
Hypothyroid	16(18.0)	0(0.0)	4(11.1)	0(0.0)	4(10.8)	0(0.0)	4(11.1)	0(0.0)
Colitis	13(14.6)	2(2.2)	4(11.1)	0(0.0)	4(13.3)	1(2.7)	4(11.1)	1(2.8)
Hyperthyroidism	9(10.1)	0(0.0)	3(8.3)	0(0.0)	3(8.1)	0(0.0)	3(8.3)	0(0.0)
Infusion related reaction	5(5.6)	0(0.0)	4(11.1)	0(0.0)	0(0.0)	0(0.0)	0(0.0)	0(0.0)
Adrenal insufficiency	4(4.5)	1(1.1)	2(5.6)	1(2.8)	0(0.0)	0(0.0)	0(0.0)	0(0.0)
Arthralgia	2(2.2)	1(1.1)	1(2.8)	1(2.8)	2(5.4)	0(0.0)	2(5.6)	0(0.0)
Hepatitis	2(2.2)	0(0.0)	2(5.6)	0(0.0)	2(5.4)	1(2.7)	2(5.6)	1(2.8)
All SAE	38(42.7)		16(44.4)		19(51.4)		19(52.8)	
SAE (not irAE)	13(14.6)		6(16.7)		12(32.4)		12(33.3)	
SAE (irAE)	29(32.6)		12(33.3)		12(32.4)		11(30.6)	
Treatment discontinuation	29(32.6)		11(30.6)		19(51.5)		18(50.0)	
Treatment-related death	2(2.2)		1(2.8)		1(2.7)		1(2.8)	

*AE*, adverse effect; *irAE*, immune-related adverse effect; *AST*, aspartate transaminase; *ALT*, alanine transaminase; *SAE*, serious adverse effect; *WBC*, white blood cell

In the pembrolizumab plus chemotherapy group, AEs related to cytotoxic anticancer agents, including hematopoietic disorders, hepatic dysfunction, renal dysfunction, fatigue, and gastrointestinal symptoms, were more frequent than that in the pembrolizumab monotherapy group, indicating that the combination regimens were more toxic than monotherapy. There were also a large number of SAEs related to chemotherapy. The frequency of immune-related AEs (irAEs) was slightly lower in the pembrolizumab plus chemotherapy group; furthermore, pneumonia and infusion reactions were less frequent.

AEs leading to treatment discontinuation were also more common in the pembrolizumab plus chemotherapy group than that in the pembrolizumab monotherapy group. There were two treatment-related deaths in the pembrolizumab monotherapy group (2.2%) and one in the pembrolizumab plus chemotherapy group (2.8%). These trends did not change after the PSM.

## Discussion

The combination of pembrolizumab plus chemotherapy or pembrolizumab monotherapy followed by chemotherapy demonstrated a similar TFS and OS for patients with advanced NSCLC exhibiting a high PD-L1 TPS. ORR and PFS were higher in the pembrolizumab plus chemotherapy group. These trends did not change after the PSM. In terms of safety, the pembrolizumab plus chemotherapy group had more AEs associated with chemotherapy and consequently had a higher frequency of treatment discontinuation and modifications.

A similar trend has been observed in previous clinical trials. The PFS with monotherapy is approximately 7 months [6, 12], and the PFS with combination therapy was 11.1 months in patients with non-squamous cell carcinoma[13] and 8.0 months in patients with squamous cell carcinoma[4]. However, these differences in PFS did not reflect long-term survival. As mentioned earlier, the 3-year survival rate is approximately 40% in clinical trials of both monotherapy and combined chemotherapy [5, 14]. Moreover, in terms of safety, in this study, the treatment discontinuation rate was comparable to that in previous studies [2, 6, 13, 15]: The treatment discontinuation rate of combination therapy was approximately 30%, and that of monotherapy was approximately 10%.

To the best of our knowledge, there is no report focusing on TFS that has examined the strategy of combination immunotherapy and chemotherapy versus immunotherapy alone. The comparison of PFS2 which was defined as progression after a subsequent therapy line or death, whichever occurs first in the past clinical trials, is not consistent with the clinical practice strategy because the number of drugs used is different since chemotherapy followed by platinum combination therapy (e.g., DTX) was administered to the experimental group. TFS has been proposed as a surrogate endpoint for OS in colon cancer where multiple drugs are used sequentially [16]. Shinno et al. suggested that TFS might be a better surrogate endpoint for OS than PFS in NSCLC, where multiple sequential drug regimens are available such as in epidermal growth factor receptor-positive lung cancer [17]. When efficacious therapies such as immunotherapy can be used as both first-line and second-line therapies, the TFS might serve as a more appropriate surrogate endpoint for OS than PFS. Although OS in this study is immature and requires further validation, we suggest that both pembrolizumab plus chemotherapy and monotherapy are valid options with similar efficacy in TFS for patients with advanced NSCLC exhibiting a high PD-L1 TPS.

This study had several limitations. First, this was a retrospective, single-center study, which might have led to bias in the regimen selection. However, the patients were not arbitrarily excluded but were consecutively enrolled and adjusted to use PSM to reduce the bias. Second, because the timing and frequency of image evaluation differed from patient to patient, subjective bias could come into play. Nevertheless, all patients were regularly followed up every one to two months at the outpatient clinic and were examined by X-ray, CT, and MRI every 3 to 6 months. Finally, some AEs were possibly overlooked because AEs were collected solely from the medical records. However, the frequency of irAEs of grade 3 or higher did not differ significantly between the previous clinical trials and the current study; the frequency was 13.2% in the KEYNOTE-024 study [14] and 14.6% in this study.

Immunotherapy is an expensive treatment, and its economic impacts need to be considered. In the USA, both monotherapy and combination therapy were suggested to be more cost-effective than chemotherapy [18, 19]; however, Barbier et al. suggested that the cost-effectiveness of combination therapy is marginal compared to monotherapy in Switzerland [20]. Moreover, combination therapy is more toxic than monotherapy. We need to select a more appropriate population for combination therapy. For example, because combination therapy exhibited a higher response rate than monotherapy, pembrolizumab plus chemotherapy could be more beneficial in patients who require a higher response, such as those with higher tumor volume and/or fast-growing tumors. Several prospective randomized studies are ongoing, and the INSIGNA trial (NCT03793179) and the NHO-Pembro-NSCLC trial (jRCTs031200078) will evaluate the efficacy of a combination of pembrolizumab and platinum + PEM for non-squamous cell carcinoma. These studies might lead to further clarity regarding these two strategies.

In conclusion, because of similar efficacy in TFS, both pembrolizumab plus chemotherapy and monotherapy would be valid options for patients with advanced NSCLC exhibiting a high PD-L1 TPS. However, combination therapy is more toxic as a first-line treatment. When efficacious therapies such as immunotherapy can be used as both first-line and second-line therapies, the TFS might serve as an appropriate surrogate endpoint for OS.

### Supplementary Information

Below is the link to the electronic supplementary material.

Supplementary file1 (PDF 339 KB)

## Data Availability

The datasets used and analyzed during the current study are available from the corresponding author on reasonable request.
