# Supplementary material for: Comparison of time to failure of pembrolizumab plus chemotherapy versus pembrolizumab monotherapy: a consecutive analysis of patients having NSCLC with high PD-L1 expression
Source: Cancer Immunol Immunother. 2021 Aug 14;71(3):737–46. doi: 10.1007/s00262-021-03029-9 (PMC8854243; doi:10.1007/s00262-021-03029-9)
Supplement: Supplementary file 1 — Supplementary file1 (PDF 339 KB) [file 262_2021_3029_MOESM1_ESM.pdf]

supplementary Table 1. Objective response rates and disease control rates

Group	Mono group (n = 89)	Mono group after PSM (n = 36)	Combo group (n = 37)	Combo group after PSM (n = 36)
CR, n (%)	2 (2.2)	1 (2.8)	0 (0.0)	0 (0.0)
PR, n (%)	37 (41.6)	17 (47.2)	25 (67.6)	25 (69.4)
SD, n (%)	18 (20.2)	5 (13.9)	10 (27.0)	9 (25.0)
PD, n (%)	23 (25.8)	10 (27.8)	2 (5.4)	2 (5.6)
NE, n (%)	9 (10.1)	3 (8.3)	0 (0.0)	0 (0.0)
ORR	43.8%	50.0%	67.6%	69.4%
DCR	64.0%	63.9%	94.6%	94.4%

CR, complete response; DCR, disease control rate; NE, not evaluable; ORR, objective response rate; PD, progression disease; PR, partial response; PSM, propensity score matching; SD, stable disease.

supplementary Table 2. Subgroup analysis of objective response rates and disease control rates

Group	Non-Sq of the Mono group (n = 70)	Sq of the Mono group (n = 19)	Non-Sq of the Combo group (n = 31)	Sq of the Combo group (n = 6)
CR, n (%)	1 (1.4)	1 (5.2)	0 (0.0)	0 (0.0)
PR, n (%)	30 (42.9)	7 (36.8)	20 (64.5)	5 (83.3)
SD, n (%)	14 (20.0)	4 (21.1)	10 (32.3)	1 (16.7)
PD, n (%)	18 (25.7)	5 (26.3)	2 (6.5)	0 (0.0)
NE, n (%)	7 (10.0)	2 (10.5)	0 (0.0)	0 (0.0)
ORR	44.3%	42.1%	64.5%	83.3%
DCR	64.3%	63.2%	96.8%	100.0%

CR, complete response; DCR, disease control rate; NE, not evaluable; Non Sq, non-squamous cell carcinoma; ORR, objective response rate; PD, progression disease; PR, partial response; PSM, propensity score matching; SD, stable disease; Sq, squamous cell carcinoma.

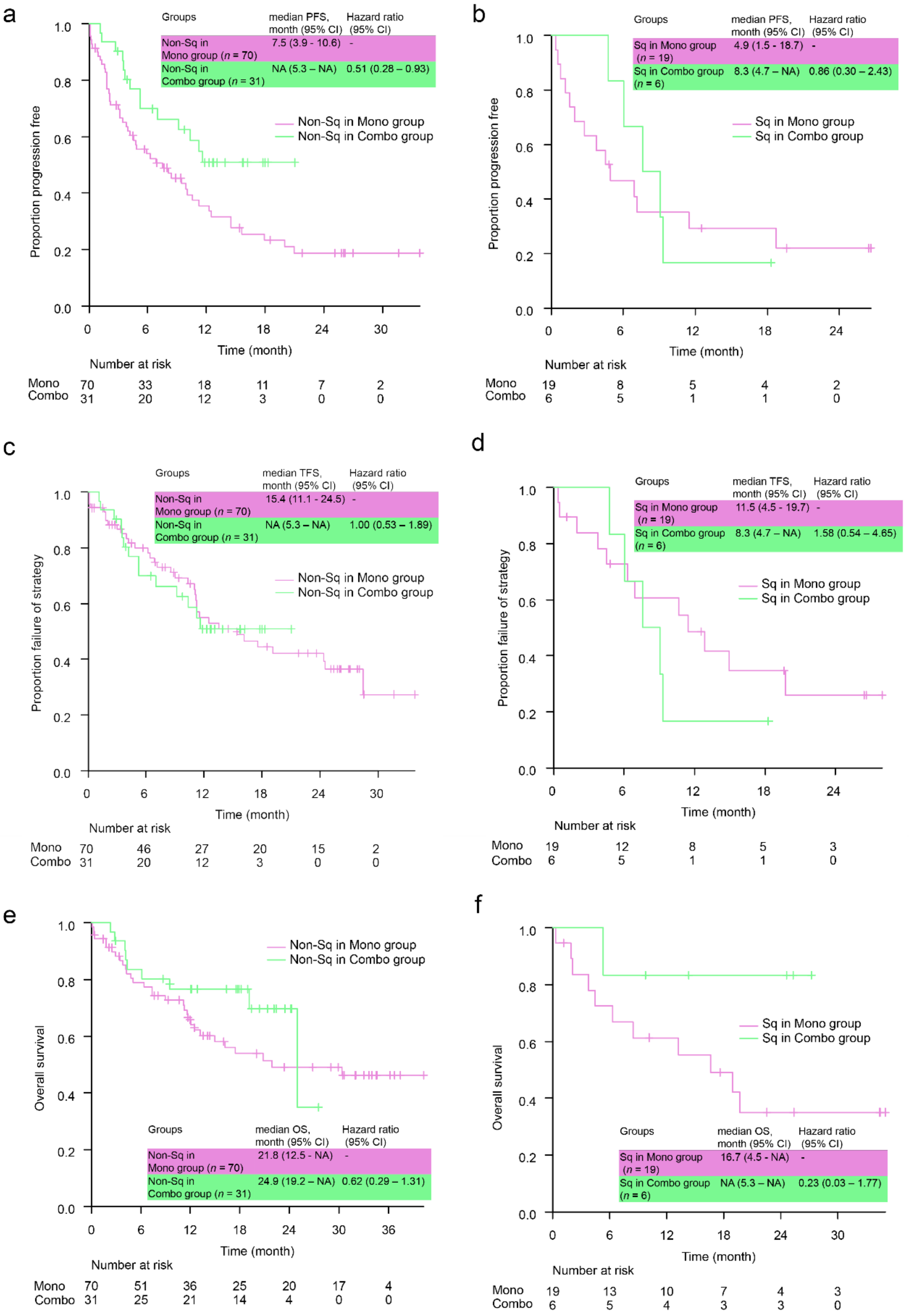

supplementary Fig.1 Subset analysis of survival curve

a. Progression-free survival of Non-Sq patients

b. Progression-free survival of Sq patients

c. Time to failure of strategy of Non-Sq patients

d. Time to failure of strategy of Sq patients

e. Overall survival of Non-Sq patients

f. Overall survival of Sq patients

PFS, TFS, and OS were estimated using the Kaplan-Maier method. The subgroup analysis did not show any significant difference in trend from the overall population.

PFS, progression-free survival; TFS, time to failure of strategy; OS, overall survival; CI, confidence interval; NA, not applicable
